# The impact of a single-nucleotide mutation of *bgl2* on cellulase induction in a *Trichoderma reesei* mutant

**DOI:** 10.1186/s13068-015-0420-y

**Published:** 2015-12-30

**Authors:** Yosuke Shida, Kaori Yamaguchi, Mikiko Nitta, Ayana Nakamura, Machiko Takahashi, Shun-ichi Kidokoro, Kazuki Mori, Kosuke Tashiro, Satoru Kuhara, Tomohiko Matsuzawa, Katsuro Yaoi, Yasumitsu Sakamoto, Nobutada Tanaka, Yasushi Morikawa, Wataru Ogasawara

**Affiliations:** Department of Bioengineering, Nagaoka University of Technology, 1603-1 Kamitomioka, Nagaoka, Niigata 940-2188 Japan; Japan Science and Technology Agency (JST), 4-1-8 Honcho, Kawaguchi, Saitama 332-0012 Japan; Department of Genetic Resources Technology, Faculty of Agriculture, Kyushu University, 6-10-1 Hakozaki, Higashi-ku, Fukuoka, 812-8581 Japan; Bioproduction Research Institute, National Institute of Advanced Industrial Science and Technology (AIST), Tsukuba Central 6, 1-1-1 Higashi, Tsukuba, Ibaraki 305-8566 Japan; School of Pharmacy, Iwate Medical University, 2-1-1 Nishitokuta, Yahaba, Iwate 028-3694 Japan; School of Pharmacy, Showa University, 1-5-8 Hatanodai, Shinagawa-ku, Tokyo, 142-8555 Japan

**Keywords:** *Trichoderma reesei*, *Hypocrea jecorina*, β-Glucosidase, Cellobiose, Cellulase induction, Gene regulation

## Abstract

**Background:**

The filamentous fungus *Trichoderma reesei* (anamorph of *Hypocrea jecorina*) produces increased cellulase expression when grown on cellulose or its derivatives as a sole carbon source. It has been believed that β-glucosidases of *T. reesei* not only metabolize cellobiose but also contribute in the production of inducers of cellulase gene expression by their transglycosylation activity. The cellulase hyper-producing mutant PC-3-7 developed in Japan has enhanced cellulase production ability when cellobiose is used as the inducer. The comparative genomics analysis of PC-3-7 and its parent revealed a single-nucleotide mutation within the *bgl2* gene encoding intracellular β-glucosidase II (BGLII/Cel1a), giving rise to an amino acid substitution in PC-3-7, which could potentially account for the enhanced cellulase expression when these strains are cultivated on cellulose and cellobiose.

**Results:**

To analyze the effects of the BGLII mutation in cellulase induction, we constructed both a *bgl2* revertant and a disruptant. Enzymatic analysis of the transformant lysates showed that the strain expressing mutant BGLII exhibited weakened cellobiose hydrolytic activity, but produced some transglycosylation products, suggesting that the SNP in *bgl2* strongly diminished cellobiase activity, but did not result in complete loss of function of BGLII. The analysis of the recombinant BGLII revealed that transglycosylation products might be oligosaccharides, composed probably of glucose linked β-1,4, β-1,3, or a mixture of both. PC-3-7 revertants of *bgl2* exhibited reduced expression and inducibility of cellulase during growth on cellulose and cellobiose substrates. Furthermore, the effect of this *bgl2* mutation was reproduced in the common strain QM9414 in which the transformants showed cellulase production comparable to that of PC-3-7.

**Conclusion:**

We conclude that BGLII plays an important role in cellulase induction in *T. reesei* and that the *bgl2* mutation in PC-3-7 brought about enhanced cellulase expression on cellobiose. The results of the investigation using PC-3-7 suggested that other mutation(s) in PC-3-7 could also contribute to cellulase induction. Further investigation is essential to unravel the mechanism responsible for cellulase induction in *T. reesei*.

**Electronic supplementary material:**

The online version of this article (doi:10.1186/s13068-015-0420-y) contains supplementary material, which is available to authorized users.

## Background

Plant cell walls comprise the most abundant biomasses on the earth and consist predominantly of cellulose. In nature, this biomass is utilized by a variety of cellulolytic organisms, which thereby play a key role in carbon recycling into the ecosystem. Among them, filamentous fungi are considered extremely efficient degraders of plant biomass and express a large amount of cellulases, which comprise three types of enzymes: cellobiohydrolases (EC 3.2.1.91) and endoglucanases (EC 3.2.1.4), which act synergistically to degrade cellulose to cello-oligosaccharides (mainly cellobiose), and β-glucosidases (EC3.2.1.21), which hydrolyze cellobiose into glucose. In addition, the recently discovered class of enzymes known as lytic polysaccharide monooxygenases (LPMOs) has been recognized as an effective auxiliary enzyme for cellulose degradation [1, 2]. *Trichoderma reesei* (an anamorph of *Hypocrea jecorina*) is a potent cellulolytic fungus that expresses a complete set of cellulases to degrade cellulose to glucose and is one of the best-studied cellulolytic fungi [3]. In addition, its remarkable ability to produce proteins has made this fungus an important commercial source of cellulases.

Generally, *T. reesei* expresses cellulase when cellulose is available as the sole carbon source. In addition to cellulose, some soluble disaccharides (cellobiose, α-sophorose, lactose, etc.) also trigger cellulase expression by this organism (reviewed by Bisaria and Mishra, and Kubicek et al. [4, 5]). Among soluble inducers, α-sophorose, a glucosyl-β-1,2-glucoside, shows the highest cellulase-inducing ability and is thought to be the transglycosylation product of β-glucosidase [6]. Under cellulase-inducing conditions, the genes encoding cellulases are transcribed coordinately. This phenomenon suggests that a common regulatory machinery controls cellulase expression [7, 8]. To date, several transcription regulators that control cellulase gene expression have been isolated from *T. reesei*. These include Xyr1, a key activator of cellulase and hemicellulase expression [9, 10], the additional activator ACEII [11] and ACEIII [12], the repressor ACEI [13, 14], and the β-glucosidase activator BglR [15]. In addition, the wide domain carbon catabolite repressor Cre1, which represses cellulase gene expression during growth on glucose, has also been reported [16–18].

Because of the industrial usefulness of *T. reesei*, cellulase hyper-secreting mutants have been isolated by systematic screening strategies involving mutagenesis mediated by UV irradiation or chemical mutagens [19]. In Japan, a *T. reesei* mutant lineage has been developed with the support of a national project. PC-3-7, a ninth generation mutant of QM6a, exhibits enhanced protein production when grown on a broad carbon source and exhibits particularly elevated cellulase induction by l-sorbose [20, 21]. Recently, it was demonstrated that PC-3-7 induces higher cellulase production on cellobiose, which had been considered a poor cellulase inducer for *T. reesei* [15].

Although factors such as the protein production ability, response to different carbon sources, and the mechanism of cellulase regulation have been analyzed in detail in PC-3-7 [8, 15, 20–30], the genetic and genomic mutation(s) underlying these phenotypes have not been determined as yet. To answer these questions, we carried out a comparative genomic analysis of the Japanese *T. reesei* mutant lineage using high-throughput genomic DNA sequencing technologies. We have identified 154 single-nucleotide polymorphisms (SNPs) between QM6a and PC-3-7 [30]. One SNP among them was found in *cre1*, encoding the carbon catabolite repressor, Cre1. Comparison of the PC-3-7 genome sequence with that of its direct parent strain, KDG-12, identified 19 genes with SNPs in the promoter, coding (leading to amino acid substitutions), or terminator regions. One of these genes encodes BglR, which is a novel fungal type Zn(II)2-Cys6 transcription regulator [15]. The effect of these SNPs in *cre1* and *bglr* on cellulase production was analyzed by a combination of gene complementation and disruption techniques [30]. Electrophoresis mobility shift assay revealed that the mutation of Cre1 affected its DNA binding property. Furthermore, from the comparison between PC-3-7 and Cre1 disruptant of PC-3-7 it was assumed that Cre1 mutation gave rise to partial release from glucose repression of cellulase gene expression. The BglR mutation brought about a partial loss of its function, which correlated with the reduced expression of β-glucosidase genes. Consequently, weakened BGL activity leads to decreased production of glucose via cellobiose hydrolysis, resulting in an indirect release from carbon catabolite repression of cellulase expression by glucose. However, it is difficult to explain why PC-3-7 expressed such an extremely high amount of protein in response to only these two mutations.

One gene among the nine genes containing a SNP leading to an amino acid substitution in PC-3-7 is the *bgl2* (*cel1a*) gene encoding the intracellular β-glucosidase, BGLII (Cel1a) [15]. BGLII belongs to the glycoside hydrolase (GH) family 1 (Carbohydrate Active Enzyme database; [31]). The *bgl2* gene has been cloned and the enzymatic property of recombinant BGLII has been characterized and it has been demonstrated that BGLII was localized in *T. reesei* cells by an over-expression experiment [32]. *T. reesei* also produces Cel3B, another intracellular GH1 β-glucosidase. Most recently, it was reported that BGLII and Cel3B play an important role in cellulase induction on lactose [33]. However, the physiological role of BGLII remains unclear with regards to the cellulase induction on cellulose. In *T. reesei* genome, there are nine genes encoding GH3 β-glucosidase [34]. It has been suggested that extracellular BGLs belonging to the GH family 3 not only hydrolyze cellobiose to glucose, but also coordinate cellulase induction to convert cellobiose into cellulase inducers such as α-sophorose by transglycosylation [35, 36]. As mentioned above, PC-3-7 has enhanced cellulase-producing ability on cellobiose and there is a SNP in *bgl2*. These facts led us to speculate that BGLII plays an important role in cellulase induction during growth on cellobiose. In the present study, we investigated the effects of *bgl2* complementation and disruption in the mutant strain PC-3-7 and the standard strain QM9414 to analyze the impact of the mutation in *bgl2* on cellulase induction.

## Results

### Single-nucleotide point mutation in *bgl2* and the resulting amino acid substitution

**Fig. 1:**
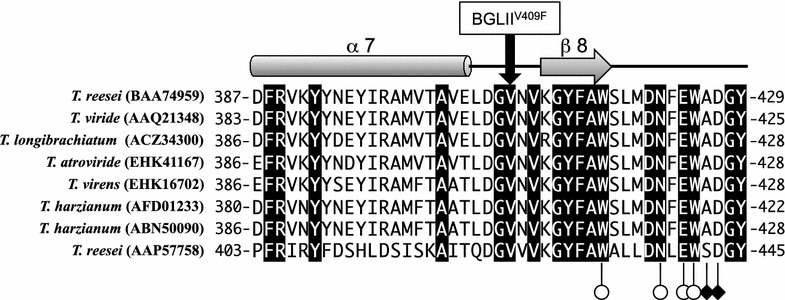
Alignment of protein sequences belonging to glycoside hydrolase family 1 from *Trichoderma*. Alignment was determined by the MUSCLE multiple sequence alignment program [60]. Conserved amino acids are indicated by a *black background*. The *T. reesei* BGLII (V409F) point mutation is indicated by the *box* at the *top*. The secondary structures of BGLII are also indicated at the *top*. The glycone and aglycone binding sites are indicated by *open circles* and *filled diamonds*, respectively [37]. The protein sequences obtained from the NCBI database are: *T. reesei* BGLII (Accession number: BAA74959), *T. viride* beta-glucosidase 2 (Accession number: AAQ21384), *T. longibrachiatum* beta-glucosidase II (Accession number: ACZ34300), *T. atroviride* glycoside hydrolase family 1 protein (Accession number: EHK41167), *T. virens* glycoside hydrolase family 1 protein (Accession number: EHK16702), *T. harzianum* 1,4-beta-glucosidase (Accession number: AFD01233), *T. harzianum* beta-1,4-glucosidase (Accession number: ABN50090), and *T. reesei* Cel1b (Accession number AAP57758)

Comparative genomic analysis of the genes encoding BGLII (*bgl2*) in KDG-12 and PC-3-7 identified a single-nucleotide difference at position 1298, where guanine was substituted by thymine. This mutation was confirmed by the sequencing of a DNA fragment comprising *bgl2* amplified by PCR using PC-3-7 genomic DNA as a template. This mutation resulted in the amino acid substitution of V409F. Figure 1 shows a multiple sequence alignment of BGLII and β-glucosidase genes from the GH family 1 of *Trichoderma* species. The amino acid corresponding to V409 of *T. reesei* BGLII is completely conserved and the amino acid sequences flanking it are also highly conserved. Based on this, we surmised that the mutation of V409F could have resulted in a change in the activity of BGLII.

### Complementation and disruption of *bgl2* in PC-3-7

In order to investigate the effect of mutation of BGLII on cellulase induction and the activity of BGLII in *T. reesei*, we constructed two types of transformants. One was a complemented strain in which mutated *bgl2* was replaced by parental *bgl2* from the QM9414 strain and the second was a *bgl2* disruptant. To transform PC-3-7, a *bgl2* recovery cassette and a *bgl2* deletion cassette were constructed and introduced into PC-3-7 with a *pyr4* and *tku70* negative background. The resulting *bgl2* revertant and *bgl2* disruptant were designated as PC-Wbgl2 and PC-∆bgl2, respectively.

**Fig. 2:**
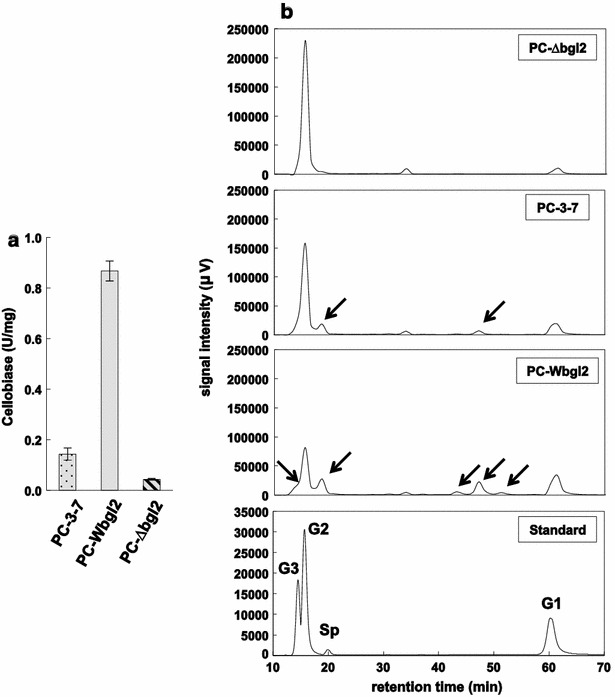
Effect of *bgl2* complementation and disruption in *T. reesei* PC-3-7. **a** Specific activity of the intracellular cellobiase from transformants PC-3-7, PC-Wbgl2, and PC-∆bgl2. Cellobiase activity represents the mean of triplicate experiments. *Error bars* indicate standard deviation. **b** HPLC analysis of transglycosylation products using cell-free extracts from transformants. In the standard chromatogram, G3, G2, and G1 represent cellotriose, cellobiose, and glucose peaks, respectively. Sp represents α-sophorose. Putative transglycosylation products are indicated by *arrows*

The strains PC-3-7, PC-Wbgl2, and PC-∆bgl2 were cultivated on medium containing Avicel for 3 days and the cellobiase activity was measured in cell-free extracts prepared from each strain. PC-Wbgl2 showed cellobiase activity that was four times that of PC-3-7, whereas PC-∆bgl2 exhibited lower activity than PC-3-7 (Fig. 2a). These results indicate that BGLII^V409F^ did not lead to a complete loss of β-glucosidase activity. To determine whether PC-Wbgl2 retained transglycosylation activity, cell-free extracts prepared from each strain were incubated under high cellobiose concentrations. Consequently, formation of transglycosylation products was observed in the presence of the PC-Wbgl2 cell-free extract. PC-3-7 extracts showed reduced activity, but some transglycosylation products were observed, whereas no such compounds were detected in the presence of PC-∆bgl2 extracts (Fig. 2b).

### Analysis of transglycosylation products by BGLII

**Fig. 3:**
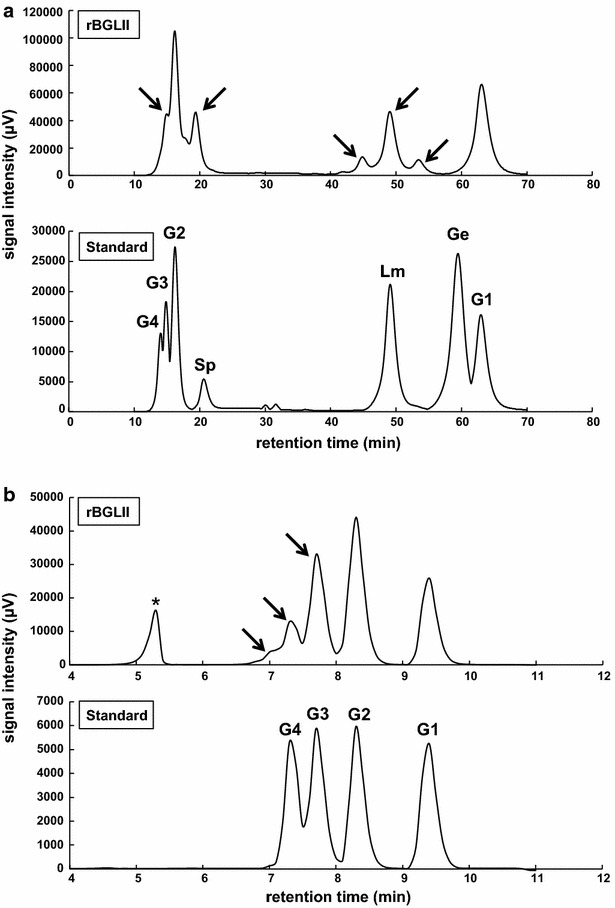
Chromatogram of transglycosylation products by rBGLIIwt. HPLC analysis of transglycosylation products using purified recombinant wild-type BGLII in *E. coli*. **a** The chromatogram generated by the Prominence HPLC system. In the standard chromatogram, G4, G3, G2, and G1 represent cellotetraose, cellotriose, cellobiose, and glucose peaks, respectively. Other β-disaccharides, α-sophorose, laminaribiose, and gentiobiose are represented by Sp, Lm, and Ge, respectively. **b** The chromatogram of size exclusion chromatography. Cello-oligosaccharides were used as the standard substance. Putative transglycosylation products are indicated by *arrows*. *Asterisk* refers to the peak of a buffer component in the reactant

**Fig. 4:**
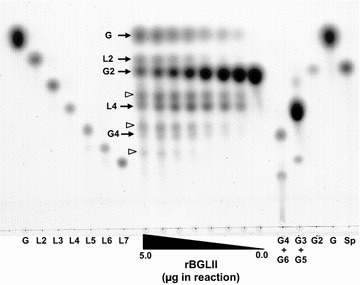
TLC Chromatogram of transglycosylation products by rBGLII. Serial amounts (5–0.075 µg by two-fold dilution) of rBGLII in 50 µL of 20 % cellobiose were subjected to TLC. The reaction mixtures were developed by silica gel plates in *n*-BuOH-AcOH-H_2_O (3:1:1, by volume) and stained with 0.5 % thymol in EtOH/H_2_SO_4_. G and Sp represent Glucose and α-sophorose, respectively. L2–7 and G2–6 represent laminari-oligosaccharides and cello-oligosaccharides. *Arrows* represent peaks consistent with the standard substance. *Open arrow* heads represent unknown transglycosylation products

The results from PC-3-7 and its transformants revealed that the formation of transglycosylation products from cellobiose was likely due to BGLII or BGLII^V409F^. However, there is also the possibility of contamination from other enzymes because we used a cell-free extract for the analysis of enzyme activity. To rule out this possibility and to measure the transglycosylation activity of BGLII exclusively, wild-type BGLII was expressed in *E. coli*. cDNA of *bgl2* was cloned into the expression vector of *E. coli*, the His-tagged recombinant wild-type BGLII, rBGLIIwt, was expressed by the induction strategy, and rBGLIIwt was purified by affinity chromatography. For the HPLC analysis of transglycosylation products of rBGLIIwt, not only glucose, cellobiose, cellotriose, and α-sophorose, but also cellotetraose, laminaribiose, and gentiobiose were used as the standard substance. HPLC data (Fig. 3a) showed that the pattern of transglycosylation products was almost the same as that from the cell-free extract of PC-3-7. In addition, the peak around 50 min of retention time was consistent with the peak of laminaribiose. To investigate the degree of polymerization of transglycosylation products, the sample was applied to a ligand exchange and size exclusion column. The analysis revealed that some kinds of oligosaccharides were present in the reactant and the larger molecules seemed to be at least tetrasaccharides (Fig. 3b). In order to analyze the transglycosylation products further, the samples were subjected to TLC together with cello-oligosaccharides, laminari-oligosaccharides, and α-sophorose as the standard material. Figure 4 shows the results of TLC in which cellobiose as the substrate was reduced and glucose as the hydrolysis product was increased according to the increase in the amount of protein in the reaction. In addition, spots having the same migration patterns as that of laminaribiose, laminaritetraose, and cellotetraose were observed. However, few spots that were not consistent with those of the standard substances were also noted.

### Cellulase production by PC-3-7, PC-Wbgl2, and PC-∆bgl2

**Fig. 5:**
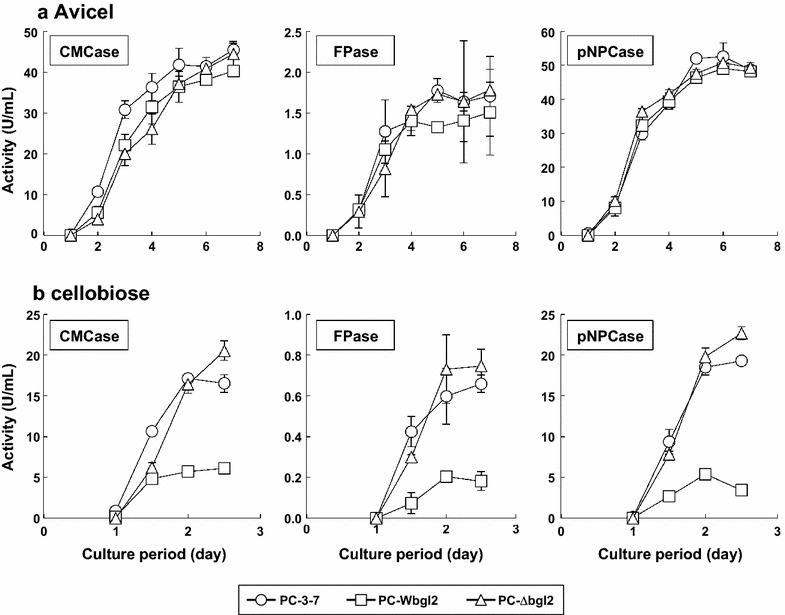
Cellulase productivity of PC-3-7, PC-Wbgl2, PC-∆bgl2. Cellulase activity of transformants grown on Avicel (**a**) or cellobiose (**b**), measured as CMCase, FPase, and pNPCase activity. As displayed in the *panel*, the *open circle* represents PC-3-7. The *open square* and the *open triangle* represent PC-Wbgl2 and PC-∆bgl2, respectively. *Values* represent the mean of triplicate experiments. *Error bars* indicate standard deviation. *X*-*axis* shows the enzyme activity per 1 mL culture medium

From the enzymatic analysis, it was evident that the SNP of *bgl2* reduced the cellobiase activity of BGLII^V409F^, which appeared to be responsible for three quarters of intracellular cellobiose degrading activity. PC-3-7 shows enhanced cellulase production on cellobiose as the sole carbon source. When *T. reesei* is cultivated on cellulose, it has been inferred that cellobiose is supplied continuously as cellulose is degraded by cellulase. Therefore, we attempted to determine whether mutation of *bgl2* affects cellulase production on cellulose or cellobiose. PC-3-7, PC-Wbgl2, and PC-∆bgl2 were grown on media containing Avicel or cellobiose and a time-course of CMCase activities was analyzed. In the case of Avicel culture, the rate of cellulase production by PC-3-7 was faster than that observed in the other two strains at the early stage of cultivation (Fig. 5a). Nevertheless, the other two strains caught up and a comparable final level of production was achieved by all three strains. This comparable cellulase activity could also be observed in the protein patterns seen on SDS-PAGE (Additional file 1: Figure S1). When transformants were grown on cellobiose, however, differences between the three strains began to emerge. PC-3-7 showed a faster production rate at the early cultivation stage similar to what we observed on the Avicel medium, but cellulase production rate of PC-Wbgl2 was significantly slower and the final activity was lower than PC-3-7, whereas PC-∆bgl2 showed slower production rate initially, but the final activity was higher than that of PC-3-7 (Fig. 5b). SDS-PAGE analysis also showed that cellulase production of PC-Wbgl2 was lower than that of PC-3-7 or PC-∆bgl2 (Additional file 1: Figure S1). These data suggest not only that SNP of *bgl2* and the resulting BGLII^V409F^ mutation affected cellulase production but that another factor(s) responsible for cellobiose signaling may also exist in PC-3-7.

**Fig. 6:**
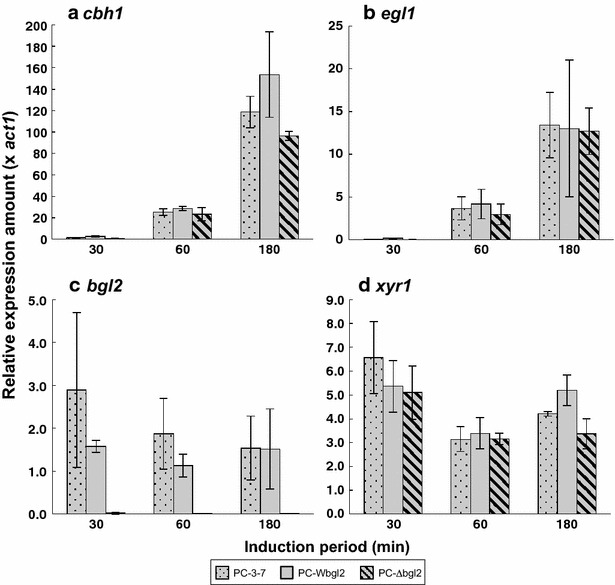
Gene expression profile of PC-3-7 transformants in response to α-sophorose induction. Genes analyzed were *cbh1* (**a**), *egl1* (**b**), *bgl2* (**c**), and *xyr1* (**d**). *Stippled gray bar* represents PC-3-7, *solid gray bar* represents PC-Wbgl2, and the *shaded gray bar* indicates PC-∆bgl2. *Values* represent the relative expression of each gene normalized to *act1* as an internal control. *Values* represent the means of triplicate experiments. *Error bars* indicate standard deviations

**Fig. 7:**
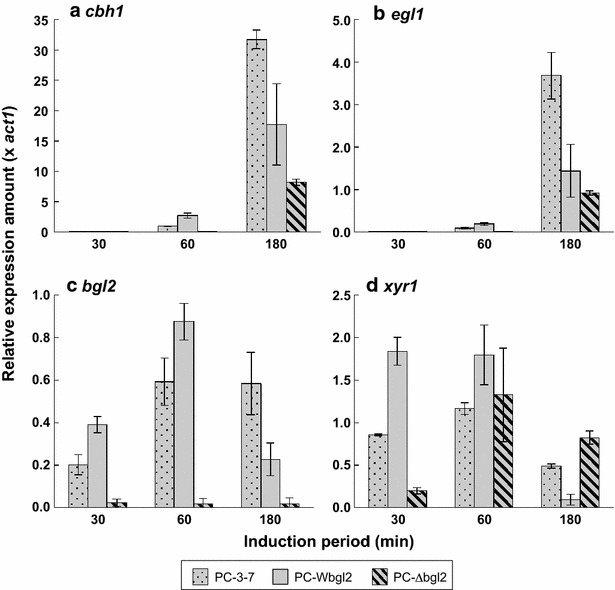
Gene expression profile of PC-3-7 transformants following cellobiose induction. Genes analyzed were *cbh1* (**a**), *egl1* (**b**), *bgl2* (**c**), and *xyr1* (**d**). Stippled gray bar represents PC-3-7, solid gray bar represents PC-Wbgl2, and the shaded gray barindicates PC-∆bgl2. Values represent the means of triplicate experiments. *Error bars* indicate standard deviations

To investigate the transcriptional response of cellulase gene toward several carbon sources without taking cell growth into account, induction experiments were carried out by using the mycelia transfer method. Each transformant was pregrown on glucose and mycelia were transferred to an induction solution containing α-sophorose, cellobiose, or Avicel as an inducer. For the target gene of expression analysis, *cbh1* and *egl1* were selected as the major cellulase genes among *T. reesei* cellulase genes that were coordinately expressed with other cellulase genes in *T. reesei*. *xyr1*, the gene encoding Xyr1 that is the key activator of *T. reesei* cellulase genes, was also chosen as the target gene. Total cDNA derived from induced mycelia was used as a template and expression amounts of *cbh1*, *egl1*, *bgl2*, and *xyr1* were determined by quantitative real-time PCR. When α-sophorose, a strong cellulase inducer for *T. reesei*, was used, the expression amounts of *cbh1* and *xyr1* were nearly the same between all three strains, although *cbh1* expression at time point of 180 min was higher in PC-Wbgl2 compared to the other two strains (Fig. 6a, d). In the case of *bgl2* expression, the level of *bgl2* of PC-3-7 was higher than that of PC-Wbgl2 at all time points (Fig. 6c). This suggests that *bgl2* expression was partly different from *cbh1* and *egl1* (Fig. 6b). As compared with α-sophorose induction, however, significant difference in gene expression pattern was observed when cellobiose or Avicel was used as an inducer. Compared to PC-3-7, the transcription of *cbh1*, *egl1*, *bgl2*, *and xyr1* of PC-Wbgl2 was higher than that of PC-3-7 at the early stages of induction (after 30–60 min) on cellobiose (Fig. 7), but the pattern was completely reversed after 180 min and PC-3-7 showed significantly higher expression than PC-Wbgl2. Similar expression pattern was observed on Avicel as well (Additional file 1: Figure S2). Contrary to cellulase production, a significant delay in transcription of all the other genes was observed in PC-∆bgl2 as compared with the other two strains in cellobiose induction (Fig. 7). Furthermore, transcription was not detected from mycelia induced on Avicel (Additional file 1: Figure S2).

### Effects of the *bgl2* mutation on *T. reesei* QM9414 strain

PC-3-7 was derived from QM9414 via five stages of mutation and screening and there are at least 154 SNPs within the promoter, coding region, and terminator regions of the genome when compared to wild-type QM6a. These SNPs and the mutations in *cre1* and *bglr* might affect cellulase production and their responses to growth on distinct carbon sources in PC-3-7, making it difficult to evaluate the effect of BGLII^V409F^ in this strain. Therefore, we chose the parental strain QM9414 with a reduced mutation background compared to that of mutants such as PC-3-7 to analyze the effects of BGLII^V409F^. The mutated *bgl2* expression cassette and the *bgl2* disruption cassette were introduced into QM9414. The resulting BGLII^V409F^ expressing strain and *bgl2* disrupted strain were called QM-Mbgl2 and QM-∆bgl2, respectively. The intracellular cellobiase activities of QM9414, QM-Mbgl2, and QM∆bgl2 following transglycosylation activities are shown in Additional file 1: Figure S3. As was the case with the transformants of PC-3-7, *bgl2* mutation and disruption resulted in a significant decrease in the intracellular cellobiase activity (Additional file 1: Figure S3A). In addition, the activity of QM-Mbgl2 was also slightly higher than that of QM-∆bgl2. Therefore, it was clear that intracellular cellobiase activity was affected by *bgl2* mutation or disruption. In terms of transglycosylation activity, despite its low level, QM-Mbgl2 produced a transglycosylation product (Additional file 1: Figure S3B).

**Fig. 8:**
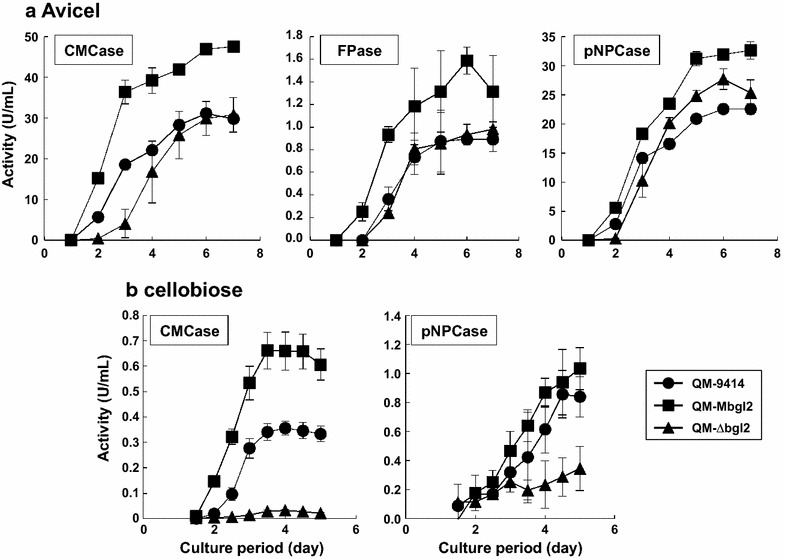
Cellulase productivity of QM9414, QM-Mbgl2, QM-∆bgl2. Cellulase activity of transformants grown on Avicel (**a**) or cellobiose (**b**), measured as CMCase FPase, and pNPCase activity. Activities of QM9414 (*filled squares*), QM-Mbgl2 (*filled circles*), and QM-∆bgl2 (*filled triangles*) are shown. *Values* represent the mean of triplicate experiments. *Error bars* indicate standard deviation. *X-axis* shows the enzyme activity per 1 mL culture medium

To investigate cellulase production in the transformants, they were cultivated on Avicel or cellobiose as the sole carbon source. When Avicel was used as the carbon source (Fig. 8a), the rate of cellulase production by QM-∆bgl2 was initially slower than that observed by QM9414, but toward the end of the culture period, the rate picked up and there was virtually no difference between the two strains with regards to cellulase production. Surprisingly, cellulase production by QM-Mbgl2 was significantly greater than QM9414 and was comparable to that observed by PC-3-7. This observation was supported by the data obtained from SDS-PAGE analysis of the culture filtrate (Additional file 1: Figure S1). Generally, cellobiose is regarded as a poor inducer of cellulase production in QM9414, and we indeed observed significantly less cellulase activity when QM9414 was cultured on cellobiose. However, when cellobiose was used as the poor cellulase inducer for QM9414, QM-Mbgl2 showed significantly more robust protein production than did QM9414 (Fig. 8b). QM-∆bgl2 exhibited significantly lower cellulase activity compared to the other two strains. In the case of FPase from QM9414 transformant, activity of each strain was under the detection limit. The cell biomass of these strains was comparable when cultivated on cellobiose, indicating the absence of any influence of the *bgl2* mutation or disruption (data not shown).

### Cellulase gene expression following culture on Avicel

**Fig. 9:**
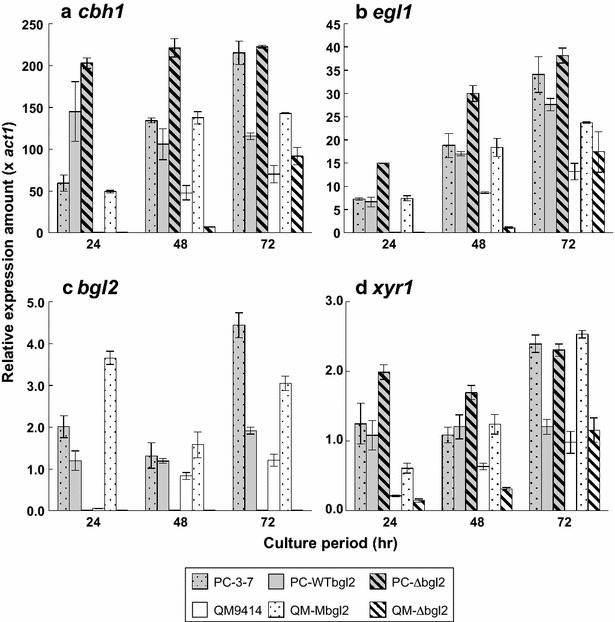
Gene expression in PC-3-7 and QM9414 transformants grown on Avicel. Genes analyzed were *cbh1* (**a**), *egl1* (**b**), *bgl2* (**c**), and *xyr1* (**d**). PC-3-7 series are expressed in *gray* and the QM-series are represented in *white*. The relative expression of each gene is normalized that of *act1* as an internal control. *Values* represent the mean of triplicate experiments. *Error bars* indicate standard deviations

To analyze cellulase gene expression in the QM-series during Avicel cultivation, quantitative real-time PCR was performed using total cDNA obtained from mycelia grown on Avicel culture as a template (Fig. 9a–d). In the QM-series, expression of all genes was greater in QM-Mbgl2 than in the other strains at all the time points examined. In the QM-∆bgl2 strain, the expression of *cbh1* and *egl1* was lower at early time points than that of QM9414, but the final level of expression was comparable to what was observed in QM9414 (Fig. 9a, b). The pattern of expression of *xyr1* in each strain derived from QM9414 (Fig. 9d) was nearly identical to the pattern of cellulase gene expression. In addition, the pattern of gene expression appeared to be similar to the cellulase production in each strain (Fig. 8a). In the case of PC-3-7, the final transcription level of cellulase gene was higher than that of PC-Wbgl2, although not at all time points. Interestingly, the expression of *cbh1* and *egl1* was the greatest in the PC-∆bgl2 strain among the PC-series. In contrast, the expression of *cbh1* and *egl1* in QM-∆bgl2 did not exceed that in QM-9414.

## Discussion

**Fig. 10:**
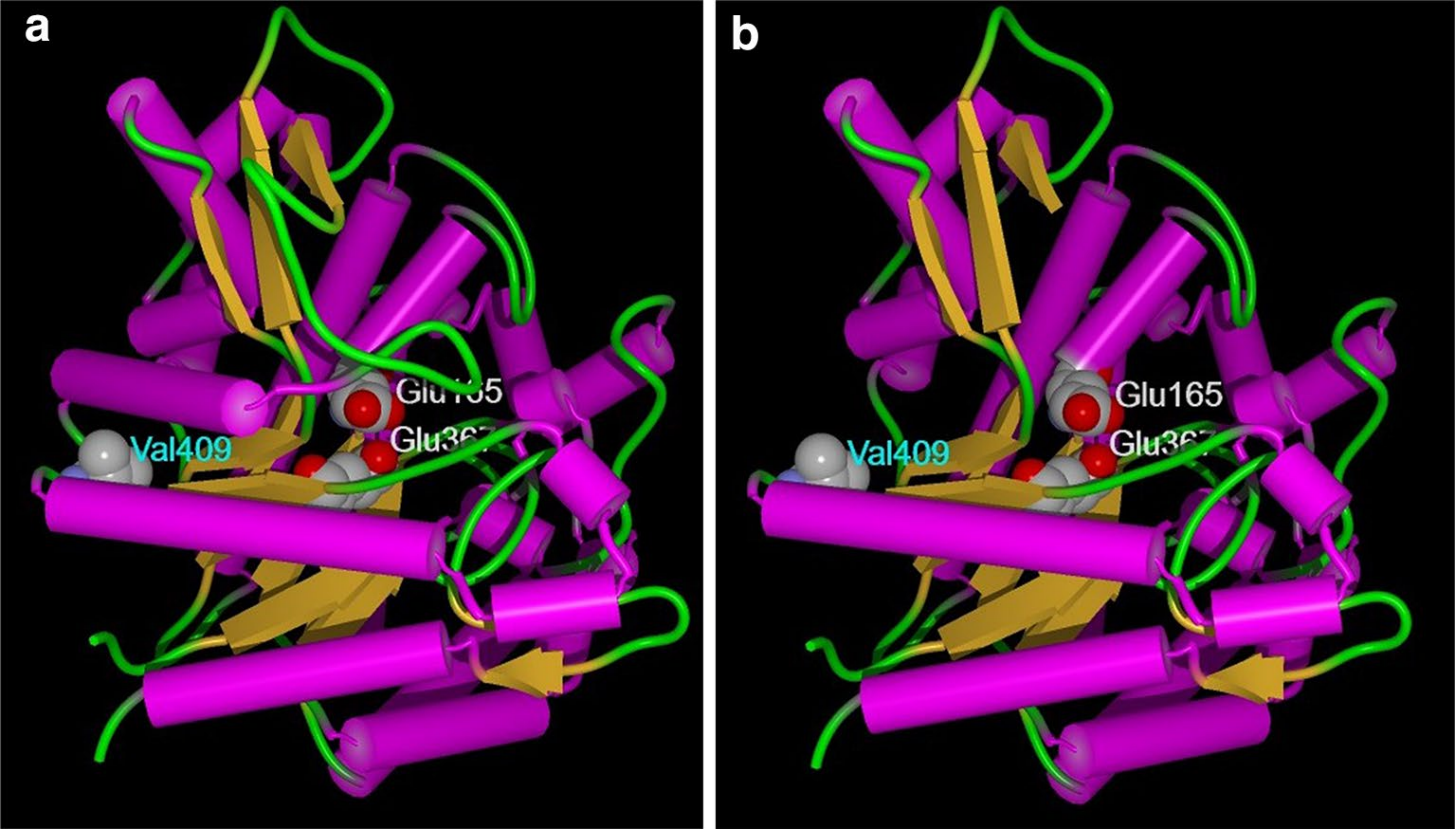
The active site and the mutation point in the three-dimensional structure of *Trichoderma reesei* BGLII (PDBID: 3AHY). The catalytic acid/base, Glu165, the catalytic nucleophile, Glu367, and the mutation residue in this study, Val409 are shown as CPK model. All other residues are displayed as ribbon model in **a** and residues from Phe323 to Tyr360 are omitted in **b**

In this study, we evaluated the effect of a single-nucleotide mutation in the *bgl2* gene of the cellulase hyper-producing mutant PC-3-7 developed in Japan on cellulase production. By comparing amino acid sequences between GH1 proteins from *Trichoderma* species, we noted that V409 was strictly conserved. V409 is located within a loop between the 7th α-helix and the 8th β-sheet. Hence, V409 is close to the glycone or aglycone recognition site [37], and substitution of V409 to phenylalanine, which is a large amino acid, could lead to the alteration of substrate recognition and enzyme activity. Through this study, we concluded that the hydrolysis activity of BGLII^V409F^ was markedly low and that transglycosylation activity, although weak, remained. From a structural point of view, V409 is localized in close proximity to the surface of the BGLII protein. However, structural analysis by a calculation method of solvent-accessible surface [38] showed that the solvent accessibility of V409 was only 1 % (Fig. 10a). If the loop and the following 6th α-helix between F323 and Y360 comprising a part of the catalytic pocket of BGLII were removed, the solvent accessibility of V409 will increase to 30 % (Fig. 10b). This means that V409 interacts with the 6th α-helix and the substitution V409F is expected to affect the static and/or dynamic structure of the loop constituting a part of the substrate pocket indirectly through the 6th α-helix. Therefore, V409 is a very interesting residue from the standpoint of the protein design with a physical perturbation method [39]. We observed reduced cellulase production by reverting the *bgl2* mutation of PC-3-7 and a significantly enhanced cellulase production when the mutation was introduced into the *bgl2* gene of strain QM9414. These facts suggest that the single-point mutation in the gene encoding BGLII^V409F^ affected cellulase expression.

In *T. reesei*, expression of cellulase genes is induced by cellulose and it has been proposed that this phenomenon is caused by the presence of trace soluble sugars derived from cellulose by constitutively expressed cellulase activity. This theory is supported by two findings: (1) trace amounts of mRNA corresponding to two major cellulase genes (*cbh1* and *cbh2*) are detected in uninduced cells and (2) the addition of anti-cellulase antibody hindered cellulase formation during growth on cellulose [40, 41]. Cellobiose is the primary end product generated from cellulose degradation by cellulases. It has been shown that cellulase production is induced by cellobiose in fungi, including *T. reesei* [42]. In some cases, cellulase formation is not observed during growth on cellobiose due to the hydrolysis of cellobiose into glucose by extracellular β-glucosidase. Glucose inhibits cellobiose uptake [43] and could give rise to carbon catabolite repression of cellulase expression. Some reports describe that the addition of a β-glucosidase inhibitor to the culture, knock-out of the major extracellular β-glucosidase, or lowering the pH of the culture medium to reduce β-glucosidase activity resulted in an enhanced cellulase production [36, 44, 45]. Since the *Km* of β-glucosidase toward cellobiose is 20–40-fold higher than that of β-glucoside permease [43], cellobiose is released from cellulose in concentrations that favor uptake rather than hydrolysis. Therefore, it is possible that cellobiose is the in vivo inducer in *T. reesei*.

The most potent inducer of cellulase gene expression is α-sophorose. It has been proposed that α-sophorose is converted from cellobiose by transglycosylation [6]. Disruption of the gene encoding the major extracellular β-glucosidase, BGLI, led to a delay in the production of cellulase on cellulose but not on α-sophorose. Previously, it has been shown that a BGLI overproducing strain showed higher cellulase production than the parental strain under non-saturating concentrations of α-sophorose. However, nojirimycin, a β-glucosidase inhibitor, inhibited cellulase production on α-sophorose [35]. Therefore, α-sophorose might not be the final inducer for cellulase induction and it is possible that some other β-glucosidase(s) are involved in true inducer formation. Recently, a comprehensive analysis of cellulase gene expression on sophorose and cellulose has been reported [46]. In this report, the authors proposed that both cellobiose and sophorose act as co-inducers of cellulase formation. Furthermore, additional compound with low molecular weight sugar, l-sorbose, promotes cellulase gene expression [8]. This suggests that several signal transduction pathways responsible for each of these inducers might control cellulase expression in parallel. Although *bgl2* expression is responsible for α-sophorose [32], it is partially released from the control of Xyr1, the xylanase and cellulase gene activator [26]. This means *bgl2* expression is controlled by some other factor on α-sophorose. Therefore, it is possible that BGLII is located upstream of the cellobiose signaling cascade in *T. reesei*. Recently, however, a report focusing on the physiological role of BGLII described that BGLII participated in the rapid induction of cellulase genes by cellulose and cellobiose [47]. In our study, a significant difference was observed in cellulase gene expression between PC-3-7 and PC-Wbgl2 strains following cellobiose induction, but not following α-sophorose induction. Cellobiase activity in cell-free extracts of the PC-3-7 strain was greatly reduced, but the transglycosylation activity still remained. An analysis of BGLII expressed in a heterologous host showed previously that it could produce α-sophorose from cellobiose or glucose [32]. In this study, we analyzed the transglycosylation products of BGLII using a recombinant enzyme. From the data of TLC (Fig. 4), the major products appeared to be Laminaribiose, laminaritetraose, and cellotetraose and not α-sophorose. In addition to these, unknown substances composed probably of three to five glucose units were also detected. These facts imply that BGLII can produce not only disaccharides but also oligosaccharides composed probably of glucose linked β-1,4, β-1,3, or a mixture of both. Further investigation is necessary to clarify the identity and characteristics of these larger molecular substances.

The cellobiose that is taken up accumulates in the fungal cell and a fraction of it is converted into α-sophorose or other substances that act as cellulase inducers. Furthermore, since glucose production from cellobiose was lowered, carbon catabolite repression by glucose might be hindered. Partial release of cellulase gene expression from glucose repression by mutation of the repressor Cre1 [30] might help to enhance cellobiose induction. This is the first report to show that the disruption of *bgl2* in *T. reesei* mutant gives rise to elevated cellulase production on cellobiose. This phenomenon suggests that the cellobiose response of PC-3-7 is affected not only by the *bgl2* mutation but also by another mutated gene(s), because there are at least 45 mutations that caused a change in amino acid sequence in the PC-3-7 genome.

PC-3-7, a derivative of QM9414, has many SNPs in its genome compared with QM6a [30]. Therefore, it was necessary to take mutations in PC-3-7 into account for analyzing the effect of *bgl2* mutation. Mutations of CRE1 that are involved in carbon catabolite repression and/or BglR related to β-glucosidase expression especially affect the cellulase gene induction in PC-3-7. In our study, we selected the common strain, QM9414, as a recipient of *bgl2* gene disruption or mutation because it was closer to the wild type and many mutants have been developed from this strain around the world [19]. Our results show that cellobiose is a weak inducer of cellulase expression for QM9414. Mutation of *bgl2* (QM-Mbgl2) led to two-fold higher cellulase production than QM9414 despite the low level of cellulase activity. QM-Mbgl2 produced comparable levels of cellulase as that of PC-3-7. It was surprising that the *bgl2* mutation would bring higher cellulase productivity to QM9414. As mentioned above, cellobiose could be an in vivo inducer in *T. reesei*. As a result, a significantly low activity of intracellular β-glucosidase activity of QM-Mbgl2 might have led to the accumulation of cellobiose in the fungal cell. In addition, the transglycosylation activity that was remaining might have caused the formation of inducers such as α-sophorose from accumulated cellobiose by transglycosylation. Previous report showed that *bgl2* disruption in QM9414 (QM-∆bgl2) results in a delay in cellulase production [47]. In our experiments, a delay in cellulase production was also observed in QM-∆bgl2 and PC-∆bgl2. However, the final cellulase production level of QM-∆bgl2 was comparable to that of QM9414, whereas the final production level of PC-∆bgl2 was equal to PC-3-7, which possessed the *bgl2* mutation, and higher than *bgl2* revertant of PC-3-7 (PC-Wbgl2). This result could be explained by the glucose produced by intracellular β-glucosidase but not BGLII. PC-3-7 was partially relieved of carbon catabolite repression but this machinery works normally in QM9414. In addition, it is also possible that the recently isolated transcription regulator BglR also aids in β-glucosidase expression, resulting in the promotion of glucose production in the cell.

In *T*. *reesei*, the key regulator of cellulase and xylanase genes is Xyr1. Previously, Derntl et al. reported a correlation between the levels of the *xyr1* and *cbh1*/*cbh2* transcripts where elevated levels of *xyr1* seem to correlate directly with the up-regulation of *cbh1* and *cbh2* transcription [48]. In this study, the expression of the cellulase genes, *cbh1* and *cbh2*, seemed to reflect *xyr1* expression in cells cultivated on cellulose. However, when Avicel induction was performed on mycelia of PC-∆bgl2 previously grown on glucose (Additional file 1: Figure S2), the expression of cellulase genes and *xyr1* was undetectable. In the cultivation experiment, conidiospores were inoculated directly into the medium. As *T. reesei* conidia contain cellulases and β-glucosidase [49, 50], it has been proposed that conidial-bound enzymes may carry out an initial degradation of cellulose. However, in the early induction stage of the transfer experiment, trace amounts of cellobiose might be released from cellulose by constitutively expressed cellulase (mentioned above). Thus, it is thought that cellobiose is taken up by the cell and converted to an inducer or hydrolyzed to glucose. When *bgl2* was deleted in PC-∆bgl2, the absence of an inducer might be the cause of the delay in PC-∆bgl2 cellulase induction. Furthermore, it is possible that cellobiose itself served as the inducer. Recently, a mutation in the Xyr1 regulatory domain was reported in a *T. reesei* mutant used in industry, which had a glucose-blind phenotype [48]. In this strain, the basal level of expression of hemicellulase and cellulase genes was elevated and the authors suggested a direct effect of glucose upon Xyr1. A functional analysis of XlnR in *Aspergillus niger* revealed the presence of a glucose inhibitory domain in the C-terminal region [51]. In addition, it has also been reported that xylose triggers the reversible phosphorylation of XlnR of *Aspergillus oryzae* [52]. If cellulase inducers (cellobiose, α-sophorose, etc.) directly interact with Xyr1, intracellular accumulation of the inducer caused by the *bgl2* mutation might represent an artificially evolved ability for a *T. reesei* mutant in which cellulase production via Xyr1 is elevated.

## Conclusion

**Fig. 11:**
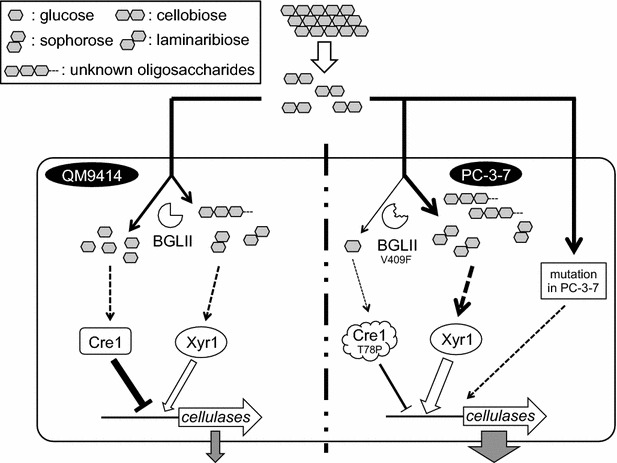
The schematic representation of the model of enhanced cellulase production responsive to cellobiose in *T. reesei* PC-3-7. Extracellular cellobioses are hydrolyzed to glucoses or transglycosylated by extracellular or cell wall-associated BGLs. Glucose causes carbon catabolite repression (CCR) of cellulase expression. However, Cre1 controlling CCR has mutated, and therefore PC-3-7 is relieved from glucose repression. Alternatively, cellobioses are incorporated in the cell and may be accumulated in the cell because BGLII, the major intracellular BGL of *T. reesei* has mutated and therefore possesses significantly reduced hydrolyzing activity. The mutated BGLII still has some residual transglycosylation activity and could generate possible cellulase inducers from cellobiose. In addition, other factor(s) that is mutated in the PC-3-7 gene through strain development from QM9414 may affect the role of cellobiose in cellulase induction. The combination of these factors might be the cause for the high cellulase production of PC-3-7

In this study, we found that the *T. reesei* mutant PC-3-7 developed in Japan possessed a mutated *bgl2* gene. Complementation of the mutated *bgl2* gene proved that this mutation was one of the means by which PC-3-7 cells acquired high cellulase expression when cultivated on cellulose and cellobiose. However, another mutation in PC-3-7 may also affect the role of cellobiose in cellulase production (Fig. 11). Furthermore, a single-nucleotide change of *bgl2* in the standard strain QM9414 brought about a positive impact upon cellulase production. Our results show not only a path to engineer *T. reesei* for industrial use, but also that mutants developed by our great predecessors have the keys to solve the regulatory puzzle of *T. reesei* cellulase induction.

## Methods

### Fungal strains and culture conditions

*T. reesei* strains QM9414 (ATCC26921) and PC-3-7 (ATCC66579) used in this study were obtained from Kyowa Hakko Bio Co., Ltd. (Tokyo, Japan). *T. reesei* strain PC-3-7 is a cellulase hyper-producing mutant with enhanced ability to respond to cellulase induction on l-sorbose [21]. Strains were grown on Difco™ Potato Dextrose Agar (PDA; BD, NJ, USA) plates and conidiospores were stored in 0.9 % NaCl solution containing 10 % glycerol until use. For cellulase enzyme production, 1 × 10^7^ conidiospores were inoculated in growth medium [20] containing Avicel cellulose (Funacel; Funakoshi、Tokyo, Japan) or cellobiose (Sigma-Aldrich, MO, USA) as the sole carbon source and were grown for appropriate durations at 28 °C on a rotary shaker (220 rpm). To carry out induction experiments using the resting cell of *T. reesei*, mycelia grown on growth medium containing glucose for 48 h were collected by filtration using Miracloth (Merck Millipore, MA, USA) and washed twice with induction medium [23] containing no carbon source. The washed mycelia were transferred to induction medium containing an appropriate carbon source and incubated for appropriate periods at 28 °C with shaking (220 rpm). After incubation, mycelia were collected as described above and frozen in liquid N_2_ for mRNA extraction.

### Construction of DNA fragments for *T. reesei* transformation

To construct the *tku70* deletion cassette, three DNA fragments were amplified by PCR. A fragment of about 3.2 kbp containing *amdS* encoding acetoamidase from *Aspergillus nidulans*, together with its upstream and downstream region was amplified using pBamdS as a template. The second fragment comprised approximately 1.6 kbp of the *tku70* upstream region and its template was the genomic DNA of PC-3-7. This fragment had a 15 bp overlap with pUC118 and the *amdS* fragment in their respective termini. The third fragment comprised approximately 1.5 kbp of the downstream region of *tku70* and introduced a 15 bp overlap with the *amdS* fragment and pUC118 in their respective termini. These three fragments and pUC118 linearized by *Eco*RI digestion were fused to create the plasmid pUdtku70S harboring the *tku70* deletion cassette by using In-Fusion Cloning Kit (Takara-Bio, Shiga, Japan).

To construct the *pyr4* disruption cassette, an approximately 4.7 kbp DNA fragment containing 2.3 kbp of the *pyr4* upstream region, the *pyr4* coding region, and the *pyr4* downstream region was amplified using sequence-specific primers. The amplicon was cloned into the *Hin*cII site of pUC118 to create pUpyr4F. Subsequently, an *Nco*I fragment within the *pyr4* coding region was removed. Finally, the plasmid pUCdpyr4 was created by self-ligation of the remaining DNA fragment. To construct another type of *pyr4* disruption cassette, an *amdS* fragment was inserted between *Aat*II sites existing within the coding region of *pyr4* of pUCdpyr4, resulting in the plasmid pUCdpyr4S.

The *bgl2* disrupting cassette and the wild-type *bgl2* expression cassette were constructed as follows: an approximately 5.3 kbp DNA fragment containing the *bgl2* coding region, with its 1.6 kbp upstream region and 2.2 kbp downstream region, was amplified with sequence-specific primers using QM9414 genomic DNA as a template. *Afl*II sites were introduced to both termini of the PCR product to excise the DNA fragment. This amplicon was inserted into the *Hin*cII site of pUC118 to obtain pUbgl2. The *pyr4* marker fragment *Sal*I-excised from pUpyr4F was inserted into the *Apa*I site within the downstream region of *bgl2*. The resulting plasmid, pUbgl2W contained the wild-type *bgl2* construct. The *pyr4* marker was inserted into *Xho*I sites existing within the *bgl2* coding and downstream regions of pUbgl2. The resulting plasmid was pUbgl2D, which contained a disrupted *bgl2*. In addition to these two plasmids, a plasmid containing a mutated *bgl2* expression cassette was constructed by site-directed mutagenesis PCR using pUbgl2W as a template. The resulting plasmid was pUbgl2M.

PCR primers used for plasmid construction are listed in Additional file 1: Table S1. PrimeSTAR^®^ HS DNA polymerase (Takara-Bio, Shiga, Japan) was used for DNA amplification. All PCR products were sequenced to confirm that there were no PCR errors.

### Transformation of *T. reesei*

Transformation of *T. reesei* PC-3-7 was performed using the protoplast-PEG method as described previously [53] with a modification in which Yatalase (Takara-Bio, Shiga, Japan) was used as a protoplasting enzyme instead of Novozyme 234 (Novozymes, Bagsværd, Denmark). *tku70* disrupting cassette released from pUdtku70S by *Eco*RI was introduced into PC-3-7. Transformed protoplasts were plated on the minimal medium containing acetoamide as the sole nitrogen source. Candidates of transformants possessing the ability to grow on acetoamide were selected and a single colony was isolated on the same medium. After single colony isolation twice, one transformant was confirmed for *tku70* disruption by Southern analysis and was named PC3-7∆k. The *pyr4* disruption cassette was excised from pUdpyr4 by *Hin*dIII and *Dra*I and introduced into PC-3-7∆k. Candidate transformants were screened on the minimum medium containing 5-flruoroorotic acid and uridine. Finally, selected uridine auxotrophic transformant was confirmed for *pyr4* disruption by southern analysis and was named PC3-7∆KP. QM9414 was transformed by the *pyr4* disrupting cassette excised from pUCdpyr4S by *Hin*dIII and *Xba*I. Candidates of transformants possessing the ability to grow on acetoamide were selected. The *pyr4* disruptant confirmed by Southern analysis was named QM9414∆P and a single colony was isolated.

To create *T. reesei* PC-3-7 strain in which *bgl2* is disrupted and wild-type *bgl2* is expressed, *bgl2* disruption cassette and wild-type *bgl2* expression cassette were excised from pUbgl2D and pUbgl2 W, respectively, by *Afl*II treatment and introduced into PC-3-7∆KP. Transformed protoplasts were plated on minimal medium without uridine to screen for cells with uridine autotrophy. Candidate transformants were streaked twice on minimal medium without uridine to obtain stable transformants. Finally, the homologous integrants with single copy of *bgl2* disruption or wild-type *bgl2* expression cassette were selected by the combination of PCR and Southern hybridization analysis (Additional file 1: Figure S4).

To create QM9414 in which *bgl2* is either disrupted or the mutant *bgl2* is expressed, the *bgl2* disruption cassette and mutant *bgl2* expression cassette from pUbgl2D and pUbgl2M, respectively, were introduced into QM9414∆P, and transformants were screened as described above.

### Preparation of recombinant BGLII in *E. coli*

The mutated *bgl2* cDNA was amplified using total cDNA derived from PC-3-7 as a template and cloned into pET22b (Merck Millipore, MA, USA) to add 6xHis-tag to the C-terminal of the expressed protein. The resulting plasmid pETbgl2F was used as a template for site-directed mutagenesis to create pETbgl2V that expresses wild-type BGLII. *E. coli* CodonPlus(DE3)-RP (Agilent Technologies, CA, USA) was transformed by pETbgl2V and used as the host of protein expression. *E. coli* harboring pETbgl2V was cultivated on the medium of Overnight Express™ Autoinduction system (Merck Millipore, MA, USA) according to the manufacturer’s instructions. After cultivation, cell lysate of harvested *E. coli* cells was prepared using xTracter Buffer (Takara-Bio, Shiga, Japan) and recombinant wild-type BGLII (rBGLIIwt) was purified by TALON^®^ Metal Affinity Resins (Takara-Bio, Shiga, Japan) as described in the instructions.

### Enzyme assays and measurement of cell biomass

Cellulase activity was determined using 1.0 % carboxymethylcellulose (CMC; Sigma-Aldrich, MO, USA), filter paper (No. 1, Whatman, LC, UK), and 4-nitrophenyl-β-d-cellobioside (pNPC; Sigma-Aldrich, MO, USA) as a substrate in 50 mM sodium acetate buffer (pH 5.0) at 50 °C for 15, 60, and 10 min, respectively. The reducing sugars produced during the enzyme reaction were determined by the Somogyi–Nelson method [54, 55] for CMCase and DNS method [56] for FPase. One unit of activity was defined as the amount of enzyme that produced 1 µmol of reducing sugars per minute in glucose equivalents. In the case of pNPCase, one unit of activity was defined as the amount of enzyme that produced 1 µmol of 4-nitrophenol. The cellobiase activity was determined in 20 mM of phosphate buffer (pH 6.5) at 45 °C with the final concentration of 20 mM of cellobiose as a substrate. The amount of glucose released by enzyme reaction was determined by Glucose C2 test Wako (Wako Pure Chemical, Osaka, Japan). One unit of cellobiase activity was defined as the amount of enzyme that produced 2 µmol of glucose per minute.

To determine cell volume, an aliquot was taken from the medium and filtered through a small piece of Miracloth. Thereafter, the mycelia were placed in a dryer for 12 h and the weight of mycelia was measured.

Protein concentration of the enzyme sample was measured by Bradford method [57] and bovine gamma globulin was used as a standard. Sodium dodecyl sulfate–polyacrylamide gel electrophoresis (SDS–PAGE) was carried out using 12.5 % polyacrylamide gel slabs as described [58]. Proteins were stained with Coomassie brilliant blue R-250 or with silver stain kit (Wako Pure Chemical, Osaka, Japan). The molecular-mass markers used were the Precision Plus Dual Standard Marker kit for SDS–PAGE (Bio-Rad Laboratories, CA, USA).

### Analysis of transglycosylation products

*Trichoderma reesei* transformants were grown on Avicel for 3 days and mycelia were disrupted using Multi Beads Shocker (Yasui Kikai, Osaka, Japan) in 50 mM of phosphate buffer (pH 6.5). Transglycosylation reaction was performed as follows. 50 µg of cell lysate were mixed in 100 µL of 20 % cellobiose in 50 mM of phosphate buffer (pH 6.5). The reaction mixture was incubated at 40 °C for 24 h. Reaction was stopped by boiling the reaction mixture in a water bath and was filtered using a membrane (0.2 µm, DISMIC^®^13HP, ADVANTEC, Tokyo, Japan). For recombinant BGLII, 100 µg of purified protein was mixed in 1000 µL of 20 % cellobiose in 50 mM of phosphate buffer (pH 6.5).

The transglycosylation reactants were analyzed by Prominence HPLC system (Shimadzu, Kyoto, Japan) equipped with Shim-Pack ISA-07/S2504 and Shim-Pack guard column ISA. A 10 µL of reactant was injected into the HPLC system with a Shimadzu SIL-20AC HT auto sampler maintained at a constant 4 °C. The column was kept at 65 °C with Shimadzu CTO-20AC column oven. Separation was carried out using a gradient of 0.1 M potassium borate buffer (pH8.0) and 0.4 M potassium borate buffer (pH 9.0) as the mobile phase at a constant flow rate of 0.6 mL/min, with 10 g/L of arginine and 30 g/L of boric acid as reaction reagents at a constant flow of 0.5 mL/min at 150 °C. Sugars were detected with RF-20AXS detector (Ex. at 320 nm, Em. at 430 nm). Glucose, cellobiose, cellotriose, cellotetraose, Laminaribiose, Gentiobiose, and α-sophorose were used as standard sugars. Transglycosylation products were also analyzed by another HPLC column, Sugar 802KS (Showa Denko, Tokyo, Japan). Separation was performed using H_2_O as the mobile phase at a constant flow 1.0 mL/min. Sugars were detected with RID-10A detector (Shimadzu, Kyoto, Japan). Glucose and cello-oligosaccharides (di- to tetrasaccharide) were used as standard sugars. For further analysis of transglycosylation products, Thin-layer chromatography was performed as described previously [59]. Transglycosylation reaction was performed using serial amounts of recombinant BGLII (5–0.075 µg by two-fold dilutions) in 50 µL of 20 % cellobiose in 50 mM of phosphate buffer (pH 6.5). Glucose, sophorose, cello-oligosaccharides (di- to hexasaccharide), and laminari-oligosaccharides (di- to heptasaccharide) were used as standard sugars.

### RNA extraction, reverse transcription, and first-strand cDNA synthesis

Total RNA was extracted from frozen mycelia by a modified hot-phenol method using TRIzol^®^ (Thermo Fisher Scientific, MA, USA) for additional purification. The lysate was applied to an RNA Spin Mini column (Buckinghamshire, UK) to remove genomic DNA and further purified following the manufacturer’s instructions. Total RNA (1 µg) was reverse-transcribed and complementary DNA was synthesized using a Transcriptor First-Strand cDNA Synthesis Kit (Roche Applied Science, Bavaria, Germany).

### Quantitative real-time PCR

Quantitative real-time PCR was performed using a LightCycler^®^ 480 System (Roche Applied Science, Bavaria, Germany). Amplification reactions were performed in a final volume of 20 µL using a LightCycler^®^ 480 SYBR Green I Master kit (Roche Applied Science, Bavaria, Germany) with 0.5 µM forward primer, 0.5 µM reverse primer, and 2 µl of cDNA (100-fold dilutant of synthesized cDNA). Thermal cycling was conducted under the following conditions: 5 min at 95 °C followed by 45 cycles of 10 s at 95 °C, 10 s at 60 °C, and 10 s at 72 °C. Assays were performed in triplicate with non-amplification controls. The specificity of the PCR amplification was documented by melting curve analysis. Relative expression levels were calculated as the log_2_ of ∆Ct value by subtracting the Ct value of the housekeeping gene (*act1*, the gene encoding actin) from that of the target gene. PCR primers used for expression analysis are listed in Additional file 1: Table S2.

## Additional file

10.1186/s13068-015-0420-y Secreted protein by transformant of PC-3-7 and QM9414. A: Avicel cultivation; 10 µL of the supernatant from day 5 of PC-3-7 series and day 6 of QM9414 series were subjected to SDS-PAGE. Gels were stained by Coomassie Brilliant Blue. B: Cellobiose cultivation; 20 µL of supernatant from day 2.5 of PC-3-7 series and day 3 of QM9414 series were subjected to SDS-PAGE. Gel of Avicel cultivation was stained by Coomassie Brilliant Blue and that of cellobiose cultivation was silver stained.   **Figure S2.** Gene expression profile of PC-3-7 transformants on Avicel induction. Genes analyzed were *cbh1* (A), *egl1* (B), *bgl2* (C), and *xyr1* (D). Stippled gray bar represents PC-3-7, solid gray bar represents PC-Wbgl2, and the shaded gray bar indicates PC-∆bgl2. Values represent the relative expression of each gene normalized to *act1* as an internal control. Values represent the means of triplicate experiments. Error bars indicate standard deviations.   **Figure S3.** Effect of bgl2 mutation and disruption in T. reesei QM9414. A: specific activity of the intracellular cellobiase from transformants QM9414, QM-Mbgl2 and QM-∆bgl2. Cellobiase activity is derived from the mean of triplicate experiments. Error bars indicate standard deviation. B: HPLC analysis of transglycosylation products using cell-free extracts from transformants. Details are as in Fig. 2. Putative transglycosylation products are indicated by arrows.   **Figure S4.** Southern analysis of transformants for *bgl2* analysis. A: the schematic representation of genomic structure of each transformants. B: results of hybridization by gene specific probe for *bgl2* and *pyr4*. Genomic DNA of each transformants was digested by SacI. Lane M represents molecular marker. **Table S1.** PCR primers used for plasmid construction. **Table S2.** primers used for real-time quantitative PCR.

## Abbreviations

BGL: β-glucosidase
SNP: single-nucleotide polymorphism
GH family: glycoside hydrolase family
PCR: polymerase chain reaction
TLC: thin-layer chromatography
SDS-PAGE: sodium dodecyl sulfate polyacrylamide gel electrophoresis
