# Supplementary material for: The impact of a single-nucleotide mutation of bgl2 on cellulase induction in a Trichoderma reesei mutant
Source: Biotechnol Biofuels. 2015 Dec 30;8:230. doi: 10.1186/s13068-015-0420-y (PMC4696228; doi:10.1186/s13068-015-0420-y)
Supplement: Supplementary file 1 — 10.1186/s13068-015-0420-y Secreted protein by transformant of PC-3-7 and QM9414. A: Avicel cultivation; 10 µL of the supernatant from day 5 of PC-3-7 series and day 6 of QM9414 series were subjected to SDS-PAGE. Gels were stained by Coomassie Brilliant Blue. B: Cellobiose cultivation; 20 µL of supernatant from day 2.5 of PC-3-7 series and day 3 of QM9414 series were subjected to SDS-PAGE. Gel of Avicel cultivation was stained by Coomassie Brilliant Blue and that of cellobiose cultivation was silver stained. Figure S2. Gene expression profile of PC-3-7 transformants on Avicel induction. Genes analyzed were cbh1 (A), egl1 (B), bgl2 (C), and xyr1 (D). Stippled gray bar represents PC-3-7, solid gray bar represents PC-Wbgl2, and the shaded gray bar indicates PC-∆bgl2. Values represent the relative expression of each gene normalized to act1 as an internal control. Values represent the means of triplicate experiments. Error bars indicate standard deviations. Figure S3. Effect of bgl2 mutation and disruption in T. reesei QM9414. A: specific activity of the intracellular cellobiase from transformants QM9414, QM-Mbgl2 and QM-∆bgl2. Cellobiase activity is derived from the mean of triplicate experiments. Error bars indicate standard deviation. B: HPLC analysis of transglycosylation products using cell-free extracts from transformants. Details are as in Fig. 2. Putative transglycosylation products are indicated by arrows. Figure S4. Southern analysis of transformants for bgl2 analysis. A: the schematic representation of genomic structure of each transformants. B: results of hybridization by gene specific probe for bgl2 and pyr4. Genomic DNA of each transformants was digested by SacI. Lane M represents molecular marker. Table S1. PCR primers used for plasmid construction. Table S2. primers used for real-time quantitative PCR. [file 13068_2015_420_MOESM1_ESM.pdf]

The impact of a single nucleotide mutation of *bgl2* on cellulase induction in a *Trichoderma reesei* mutant

Yosuke Shida[a], Kaori Yamaguchi[a], Mikiko Nitta[a, b], Ayana Nakamura[a], Machiko Takahashi[a], Shun-ichi Kidokoro[a], Kazuki Mori[c], Kosuke Tashiro[c], Satoru Kuhara[c], Tomohiko Matsuzawa[d], Katsuro Yaoi[d], Yasumitu Sakamoto[e], Nobutada Tanaka[f], Yasushi Morikawa[a], †Wataru Ogasawara[a]

[a]Department of Bioengineering, Nagaoka University of Technology, 1603-1 Kamitomioka, Nagaoka, Niigata, 940-2188, Japan, [b]Japan Science and Technology Agency (JST), 4-1-8 Honcho, Kawaguchi, Saitama 332-0012, Japan, [c]Department of Genetic Resources Technology, Faculty of Agriculture, Kyushu University, 6-10-1 Hakozaki, Higashi-ku, Fukuoka, 812-8581, Japan, [d]Bioproduction Research Institute, National Institute of Advanced Industrial Science and Technology (AIST), [e]School of Pharmacy, Iwate Medical University, 2-1-1 Nishitokuta, Yahaba, Iwate, 028-3694, Japan, Tsukuba Central 6, 1-1-1 Higashi Tsukuba, Ibaraki, 305-8566, Japan, [f]School of Pharmacy, Showa University, 1-5-8 Hatanodai, Shinagawa-ku, Tokyo 142-8555, Japan

Corresponding author (†) is Dr. Wataru Ogasawara

Nagaoka University of Technology, 1603-1 Kamitomioka, Nagaoka, 940-2188, Japan

Tel: +81-0258-47-9429

Fax: +81-0258-47-9429

E-mail: owataru@vos.nagaokaut.ac.jp

Supplementary information includes:

Supplementary Figure S1-S4

Supplementary Tables S1-S2

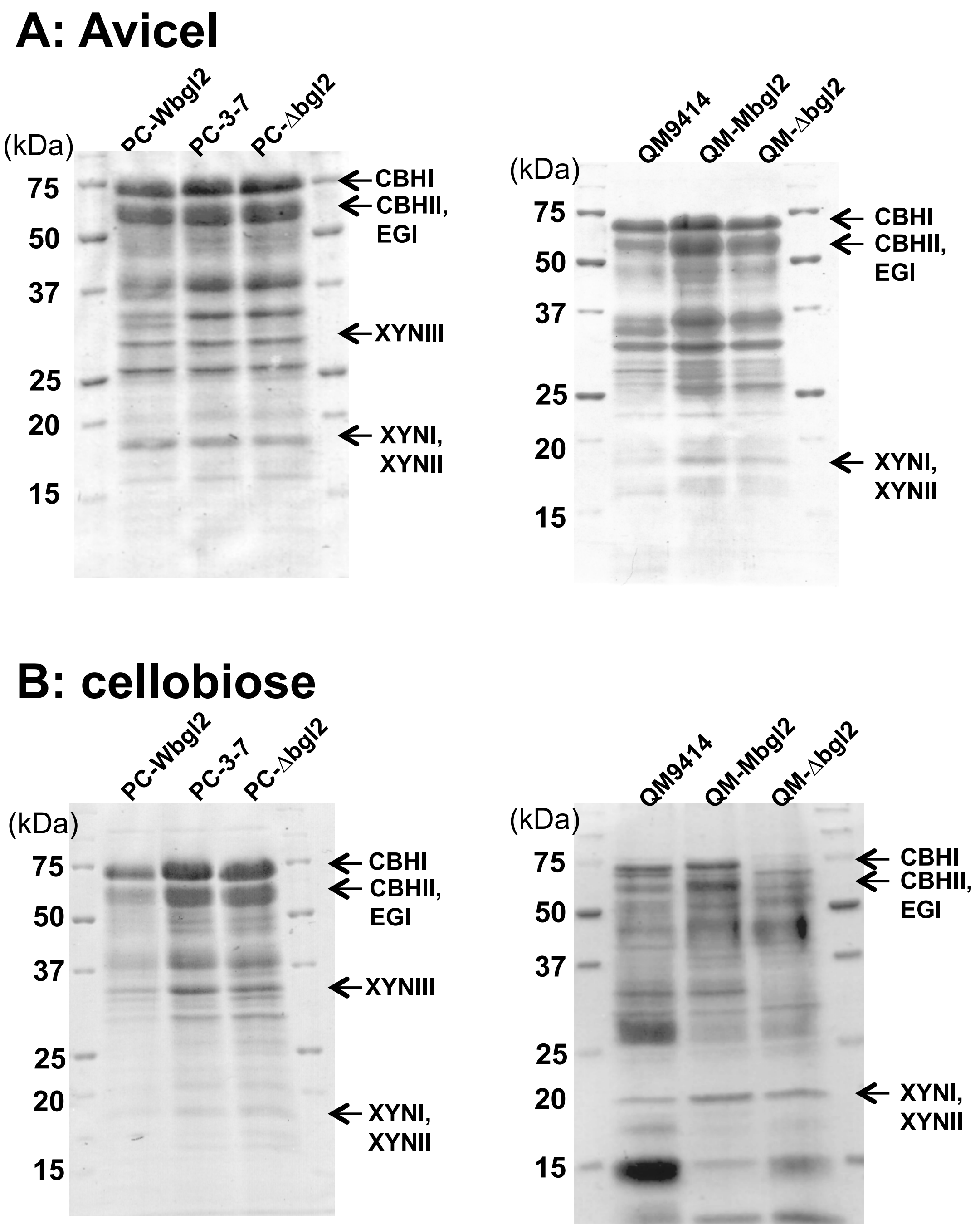

Figure S1 Secreted protein by transformant of PC-3-7 and QM9414

A: Avicel cultivation; 10 μL of the supernatant from day 5 of PC-3-7 series and day 6 of QM9414 series were subjected to SDS-PAGE. Gels were stained by Coomassie Brilliant Blue.

B: Cellobiose cultivation; 20 μL of supernatant from day 2.5 of PC-3-7 series and day 3 of QM9414 series were subjected to SDS-PAGE. Gel of Avicel cultivation was stained by Coomassie Brilliant Blue and that of cellobiose cultivation was silver stained.

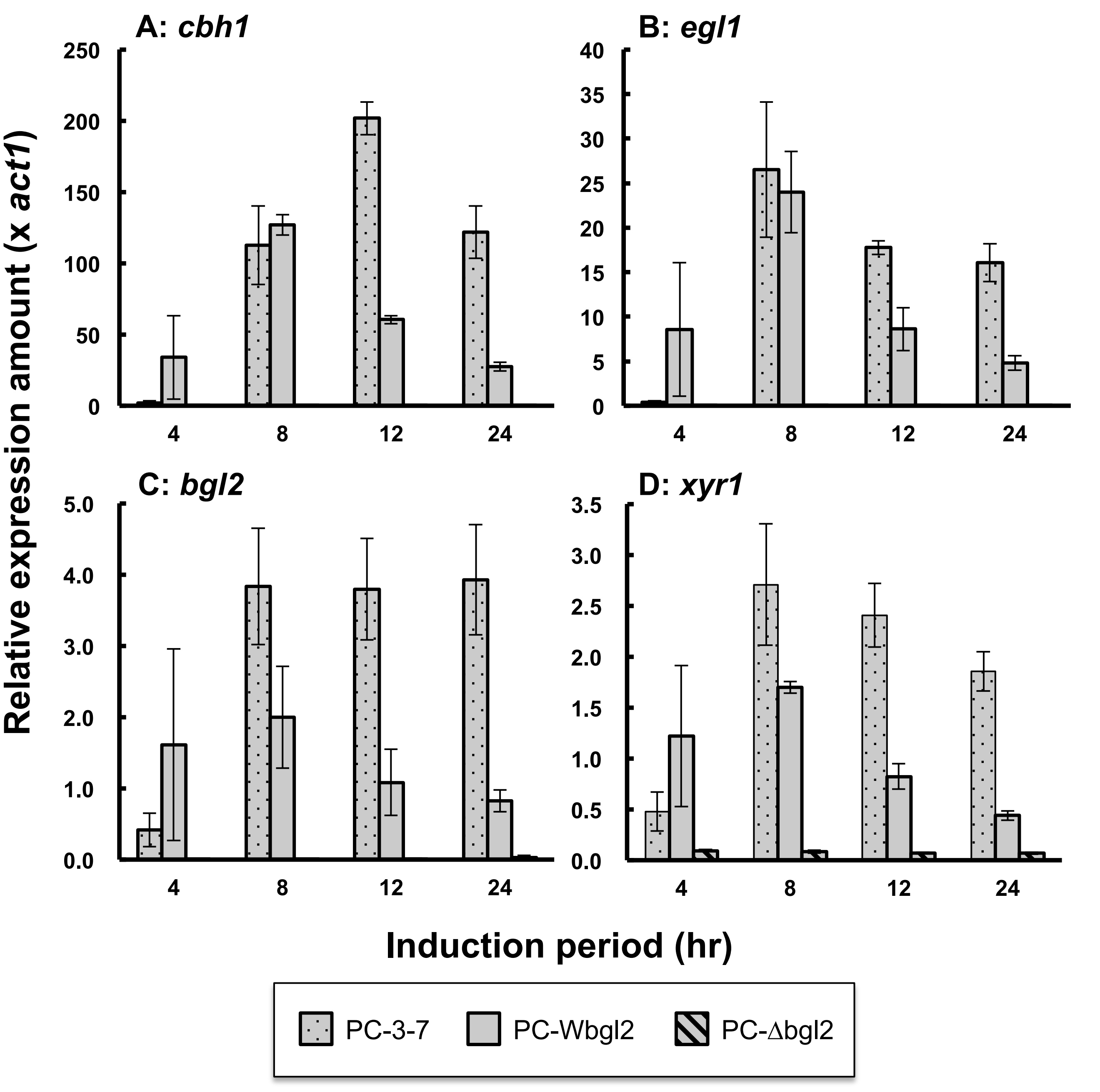

Figure S2 Gene expression profile of PC-3-7 transformants on Avicel induction.

Genes analyzed were *cbh1* (A), *egl1* (B), *bgl2* (C), and *xyr1* (D). Stippled gray bar represents PC-3-7, solid gray bar represents PC-Wbgl2, and the shaded gray bar indicates PC-Δbgl2. Values represent the relative expression of each gene normalized to *act1* as an internal control. Values represent the means of triplicate experiments. Error bars indicate standard deviations.

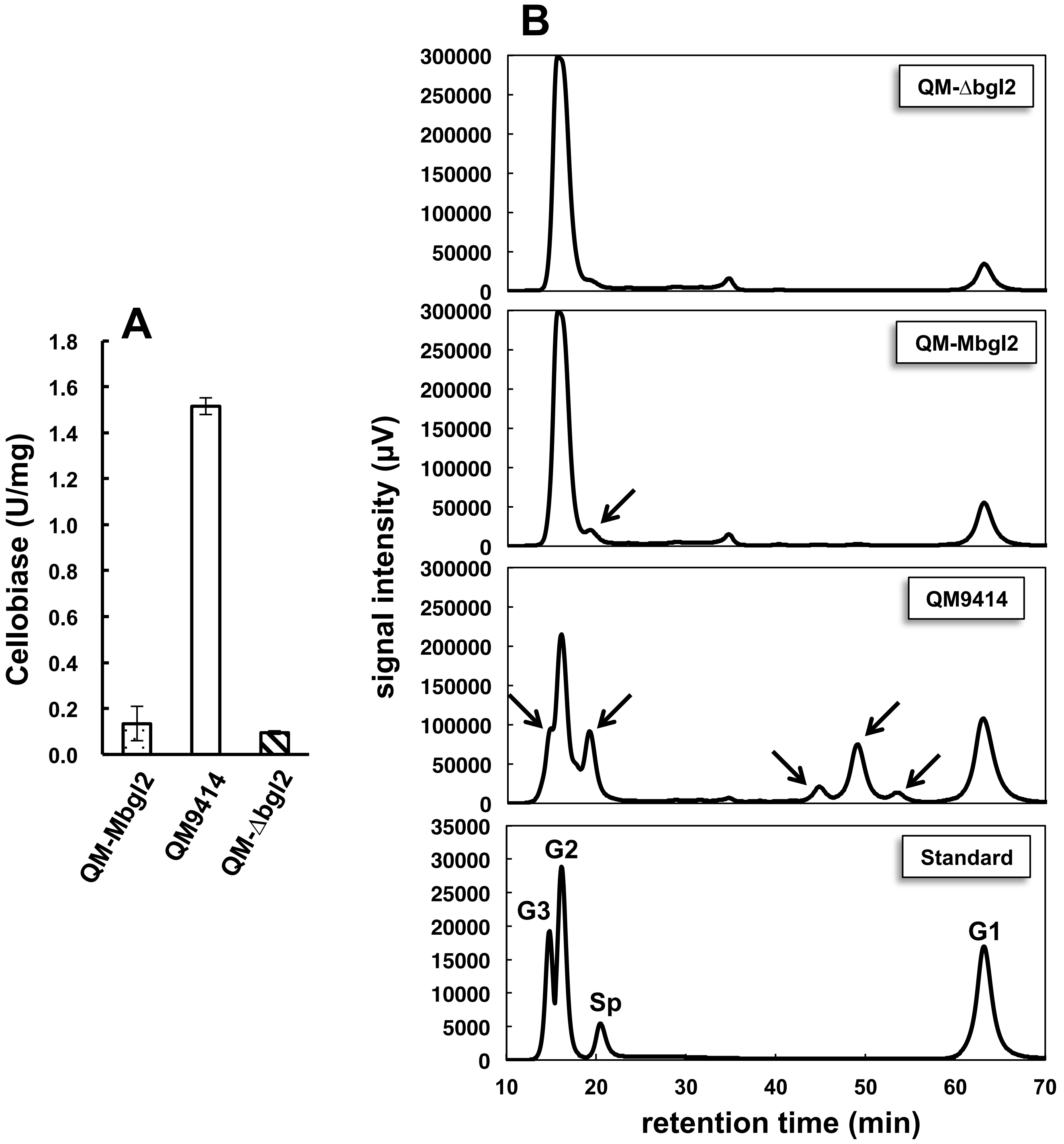

Figure S3 Effect of bgl2 mutation and disruption in *T. reesei* QM9414.

A: specific activity of the intracellular cellobiase from transformants QM9414, QM-Mbgl2 and QM-Δbgl2. Cellobiase activity is derived from the mean of triplicate experiments. Error bars indicate standard deviation. B: HPLC analysis of transglycosylation products using cell-free extracts from transformants. Details are as in Figure 2. Putative transglycosylation products are indicated by arrows.

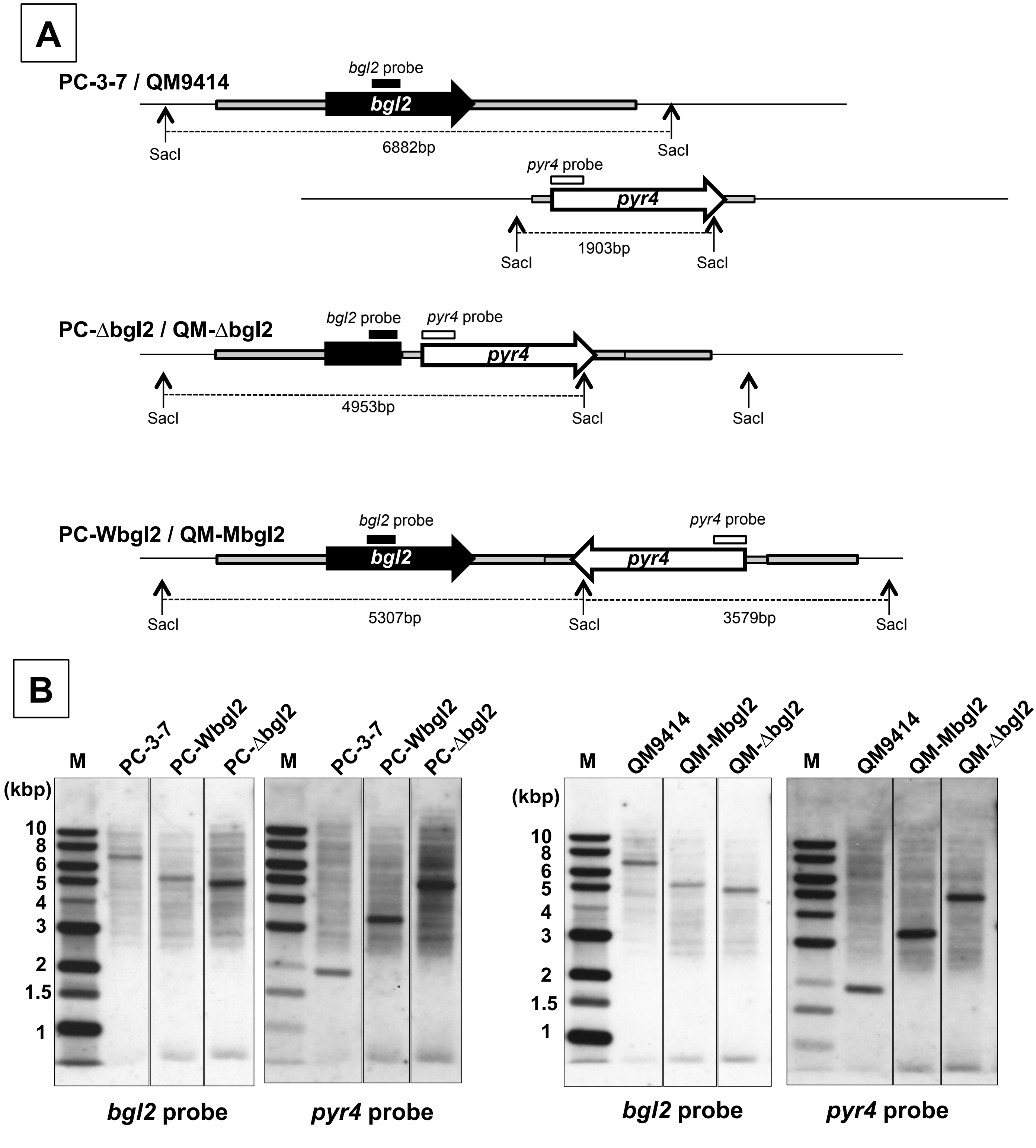

Figure S4 Southern analysis of transformants for *bgl2* analysis.

A: the schematic representation of genomic structure of each transformants.
B: results of hybridization by gene specific probe for *bgl2* and *pyr4*. Genomic DNA of each transformants were digested by SacI. Lane M represents molecular marker.

Table S1 PCR primers used for plasmid construction

primer	sequence (5' to 3')	description
tku70upFw	catgattacgaattcTGCCGTTCAAGCTGTCCACCCGCT	amplification of *tku70* upstream region (lower case: identical to pUC118)
tku70upRv	gcctatccaatgatgCAAACGCGACGAGCCTAGGCAGAAT	amplification of *tku70* upstream region (lower case: identical to *amdS*)
amdSFw	CATCATTGGATAGGCAGATTACTCAGCCTG	amplification of *tku70*
amdSRv	CTGGAAACGCAACCCTGAAGGGATTCTTCC	
tku70downFw	gggttgcgtttccagGCATTCACTACCTTGATGCTGTCGG	amplification of *tku70* downstream region (lower case: identical to *amdS*)
tku70downRv	accgagctcgaattcTTTGGGCAGCCGCTTGTCCAGCTTG	amplification of *tku70* downstream region (lower case: identical to pUC118)
bgl2Fw	TGCcttaagGCGTAACTCGATAGCA	*bgl2* amprification (lower case: AflII site)
bgl2Rv	GCGAAGCTCGATcttaagTATC	
bgl2mutFw	GGACGGGtTCAACGTCAAGGGGTACTTTGCCTG	*bgl2* mutagenesis (lowercase: substituted base)
bglmutRv	ACGTTGAaCCCGTCCAGCTCCACGGCGGTAACC	

Table S2 primers used for real-time quantitative PCR

target gene	forward primer (5' to 3')	reverse primer (5' to 3')
act1	TCCATCATGAAGTGCGAC	GTAGAAGGAGCAAGAGCAGTG
cbh1	CTTGGCAACGAGTTCTCTT	TGTTGGTGGGATACTTGCT
egl1	CGGCTACAAAAGCTACTACG	CTGGTACTTGCGGGTGAT
bgl2	CGTGCTCTTCACCAACAA	TCTTGCTGATCCACACCA
xyr1	TGCGAGACCATTGTTAGG	CTGCTGCTCAGCTAAATCTT
